# UAV Localization Algorithm Based on Factor Graph Optimization in Complex Scenes

**DOI:** 10.3390/s22155862

**Published:** 2022-08-05

**Authors:** Jun Dai, Songlin Liu, Xiangyang Hao, Zongbin Ren, Xiao Yang

**Affiliations:** 1 Institute of Geospatial Information, Information Engineering University, Zhengzhou 450001, China or; 2 School of Aerospace Engineering, Zhengzhou University of Aeronautics, Zhengzhou 450001, China; 3 Dengzhou Water Conservancy Bureau, Dengzhou 474150, China

**Keywords:** UAV, multi-source fusion, factor graph optimization, robustness

## Abstract

With the increasingly widespread application of UAV intelligence, the need for autonomous navigation and positioning is becoming more and more important. To solve the problem that UAV cannot perform localization in complex scenes, a new multi-source fusion framework factor graph optimization algorithm is used for UAV localization state estimation in this paper, which is based on IMU/GNSS/VO multi-source sensors. Based on the factor graph model and the iSAM incremental inference algorithm, a multi-source fusion model of IMU/GNSS/VO is established, including the IMU pre-integration factor, IMU bias factor, GNSS factor, and VO factor. Mathematical simulations and validations on the EuRoC dataset show that, when the selected sliding window size is 30, the factor graph optimization (FGO) algorithm can not only meet the requirements of real time and accuracy at the same time, but it also achieves a plug-and-play function in the event of local sensor failures. Finally, compared with the traditional federated Kalman algorithm and the adaptive federated Kalman algorithm, the positioning accuracy of the FGO algorithm in this paper is improved by 1.5–2-fold, and can effectively improve autonomous navigation system robustness and flexibility in complex scenarios. Moreover, the multi-source fusion framework in this paper is a general algorithm framework that can satisfy other scenarios and other types of sensor combinations.

## 1. Introduction

With the increasing demand for unmanned, intelligent, and autonomous features in various fields, UAVs (unmanned aerial vehicles) perform well in high-risk, complex, and repetitive tasks, so this has become a research hotspot and has developed rapidly. Whether in the military field or in civilian applications, UAV technology has higher and higher requirements for the accuracy of the navigation system [1,2]. A single navigation method cannot meet the requirements of UAV navigation accuracy and robustness. In complex scenes, combined and multi-source fusion navigation methods can provide more accurate and robust navigation and positioning results [3,4].

Multi-source sensor information fusion algorithms include the weighted average method, maximum likelihood estimation, least squares, Kalman filtering, D-S evidence theory, FGO, etc. [4]. In a complex environment, because some sensors are prone to failure, the system is required to realize asynchronous heterogeneous navigation source data fusion with the plug-and-play function. Most of the traditional multi-sensor information fusion methods use the integrated navigation method based on a federated Kalman filter (FKF). Although this method can fuse sensor information of different rates and calculate the navigation solution in real time through the data synchronization processing method, it is often necessary to discard a part of the measured values in order to maintain synchronization of the data, which will result in a waste of information. At the same time, the standard Kalman filter can only solve linear problems, and most sensor models contain nonlinear components [5,6].

The multi-sensor information fusion navigation algorithm based on a factor graph can solve the problems of traditional methods and has the advantage of plug and play. The fusion framework based on the factor graph model can effectively solve the asynchronous problem in data fusion, which has good scalability for multi-sensors and can be flexibly configured for sensors [7,8,9,10].

In 2007 and 2010, Dr. Levinson [11,12] of Stanford University proposed a method based on factor graph optimization to achieve map-based high-precision positioning. Ding et al. [13] made a major change to the data fusion framework, from filtering to factor graph optimization. Pfeifer et al. [14] uses a Gaussian mixture model to model the GNSS error model, and the final positioning accuracy is significantly improved.

Wang et al. [15] conducted research on the key technologies of all-source navigation and constructed a multi-sensor fusion framework based on factor graphs. The sensors involved include IMU, GPS, barometric altimeter, and optical flow sensors. Aiming at the collaborative navigation problem of densely clustered UAVs, Chen et al. [16] proposed a method for the collaborative navigation of UAV swarms based on factor graph optimization. The simulation test results show that the proposed method can effectively improve the positioning accuracy in multi-UAV application scenarios such as dense swarms. Tang et al. [17] defined the IMU, BDS, and odometer measurement factors, and constructed a multi-sensor fusion framework based on factor graphs. Gao et al. [18] constructed a factor graph model of the INS/GNSS/OD integrated navigation system, which can continuously and stably output high-precision navigation results, meeting the requirements of the vehicle-mounted INS/GNSS/OD system. Indelman et al. [19] demonstrated the proposed method in a simulated environment using IMU, GPS, and stereo vision measurements, and compared it to the optimal solution obtained by a full non-linear batch optimization, and to a conventional extended Kalman filter (EKF).

The above literature all focus on the establishment of the basic model and framework of the factor graphs. In order to study the asynchronous fusion problem of multi-source sensors, Xu et al. [20] proposed a multi-sensor information fusion method based on a factor graph, to fuse all available asynchronous sensor information, and to efficiently and accurately calculate a navigation solution. Considering the robustness of complex scenarios, Wei et al. [21] constructed an INS/GPS/OD factor graph model using factor graph technology, designed a dynamic weight function, and adjusted the weight of each factor reasonably and dynamically, thereby improving the navigational performance and robustness of the factor graph algorithm.

Artificial general intelligence (AGI) is a critical method to solve the problem that UAV cannot perform localization in complex scenes. With the increasingly widespread application of UAV intelligence, the need for autonomous navigation and positioning is becoming more and more important. AGI is a complementary approach for the factor graph optimization algorithm. Spiking Neural Networks (SNNs) are a way to solve problems such as high energy consumption in current machine learning techniques. Yang et al. [22] proposed a new spiking-based framework with minimum error entropy called MeMEE, which uses entropy theory. A gradient-based online meta-learning scheme is constructed in a recurrent SNN architecture to improve the applicability of a spike-based online meta-learning model for robust learning based on spatiotemporal dynamics and excellent machine learning theory. Aiming at the problem that there is a large gap between the learning performance of SNN and artificial neural network when there are few samples, Yang et al. [23] proposed Heterogeneous Ensemble-based Pulse-Driven Few-Shot Online Learning (HESFOL), which uses entropy theory to build a recurrent SNN architecture based on the gradient few-shot learning scheme, thereby improving the ability of SNN few-shot learning. In the field of text recognition, textual information has been shown to play an active role in recommender systems. Liu et al. [24] propose an Adaptive Attention Module (SAM), which reduces the potential selection bias caused by textual information by adjusting selection bias by capturing contextual information according to its representation. Aiming at the credit assignment problem of adjusting the weights of each neuron based on network routing error information in neuromorphic computing, Yang et al. [25] proposed a novel dendritic event-based processing (DEP) algorithm, which uses two dendrites with partially separated dendrites. Chamber leakage integrates and fires neurons, effectively solving the credit assignment problem.

It can be seen from the above, that compared with the filtering algorithm, factor graph optimization inherits the idea of iteratively finding the optimal solution in graph optimization and has a higher precision. Compared with the traditional optimization algorithm, the factor graph optimization adopts the incremental reasoning algorithm, which has strong real-time performance [26,27]. The FGO can better solve the nonlinear problem of some state equations and observation equations in the navigation system, which lays the foundation for the realization of high-precision and robust positioning and navigation technology [28,29,30,31].

However, most of the previous studies are the comparison of the factor graph optimization algorithm and FKF, and a lack of comparison with other improved FKF algorithms. Moreover, most of these studies focus on factor graph optimization algorithms for state estimation, which do not consider the effect of window size on accuracy and real-time performance. In this paper, we start from the general adaptability of the algorithm, and use the data set collected in actual experiments to verify the algorithm. Further considering the adjustment of the sliding window, the optimal window size is chosen to balance accuracy and time. Specifically, we make the following contributions.

We propose a method for navigating and localizing UAVs using factor graphs for state estimation. By analyzing the IMU pre-integration factor, IMU (inertial measurement unit) bias factor, GNSS (Global Navigation Satellite Systems) factor, and the VO (visual odometry—where the position and attitude data are obtained by solving camera image poses) factor model—the IMU/GNSS/VO factor graph framework is constructed.

We perform two types of experiments to verify the effectiveness of the proposed factor graph framework in different scenarios, including mathematical simulation experiments and EuRoC datasets.

We balance time and accuracy by setting the size of the sliding window. By comparing the state estimation results of the factor graph, the federated Kalman filter, and the adaptive Kalman filter, the robustness of the factor graph algorithm in this paper is verified.

The rest of this paper is organized as follows: The factor graph model and the iSAM incremental inference algorithm are introduced in Section 2. In Section 3, a factor graph multi-source fusion model framework based on IMU/GNSS/VO is established. In Section 4, the traditional federated Kalman filter and the adaptive federated Kalman are listed for the comparative analysis of subsequent mathematical simulation experiments. In Section 5, through mathematical simulation experiments, the accuracy performances of the three algorithms are discussed and analyzed on the basis of the selected sliding window size. In Section 6, the effectiveness of the FGO algorithm is further demonstrated through dataset validation. Finally, conclusions and further research arrangements are drawn in Section 7.

## 2. Factor Graph Model

### 2.1. Factor Graph Algorithm

The factor graph model [7,32,33,34] is expressed as (1).

(1) G=(F,X,E)

where X represents a set of variable nodes, F represents a set of factor nodes, and E represents a set of all edges connecting nodes.

The purpose of the factor graph optimization algorithm is to find the maximum posterior estimated probability.

(2) $$\hat{X}=\underset{X}{\arg\;\max}\underset{k,i}{\Pi }P(Z_{k,i}|X_{k})\underset{k}{\Pi }P(X_{k}|X_{k-1},u_{k})$$

where $Z_{k,i}$ represents the observed value, $\hat{X}$ represents the maximum posterior estimate, $P(Z_{k,i}|X_{k})$ represents the observed probability density, and $P(X_{k}|X_{k-1},u_{k})$ represents the prior probability density.

By equivalent conversion to the solution factor sum product, a factor graph is thus defined as a decomposition of the function $f_{i}(X_{i})$ of the population of variables. The following Equation (3) is obtained.

(3) $$f(X)=\underset{i}{\Pi }f_{i}(X_{i})$$

The factor graph algorithm is the optimal estimated value of the solution, so that the function takes the maximum value.

(4) $$\hat{X}=\underset{X}{\arg\;\max}(\underset{i}{\Pi }f_{i}(X_{i}))$$

The error model cost function is defined as (5).

(5) $${\left\|e\right\|}_{\Sigma }^{2}\overset{\Delta }{=}\exp(-\frac{1}{2}{\left\|h_{i}(X_{i})-z_{i}\right\|}_{\Sigma_{i}}^{2})$$

where $h_{i}(X_{i})$ is the observation function. Assuming that the above noise is a Gaussian noise model, ${\left\|e\right\|}_{\Sigma }^{2}=e^{T}\Sigma^{-1}e$ represents the Mahalanobis distance, Σ represents the covariance matrix, and (5) is transformed into (6) as follows.

(6) $$f_{i}(X_{i})\propto \exp(-\frac{1}{2}{\left\|h_{i}(X_{i})-z_{i}\right\|}_{\Sigma_{i}}^{2})$$

To sum up, (4) is transformed into a standard nonlinear least squares problem such as (7), whereby the optimal state value of the factor graph is obtained by optimizing the minimized error function, as follows.

(7) $$\hat{X}=\underset{X}{\arg\;\min}(\Sigma_{i}{\left\|h_{i}(X_{i})-z_{i}\right\|}_{\Sigma_{i}}^{2})$$

### 2.2. Incremental Smoothing and Mapping (iSAM)

The factor graph model is a dynamic graph model that increases with time. Considering the real-time requirements, the method of iSAM should be adopted.

Equation (7) is expanded by Taylor to obtain a linear least squares problem, and the state update variables are as follows.

(8) $$\delta^{*}=\underset{\delta }{\arg\;\min}{\left\|H_{i}\delta_{i}-\{z_{i}-h_{i}(X_{i}^{0})\}\right\|}_{\Sigma_{i}}^{2}$$

where $\delta_{i}=X_{i}-X_{i}^{0}$ represents the state update vector, $H_{i}={\left.\frac{\partial h_{i}(X_{i})}{\partial X_{i}}\right|}_{X_{i}^{0}}$ represents the Jacobian measurement, $\delta^{*}$ represents a local linear solution, and $z_{i}-h_{i}(X_{i}^{0})$ is the difference between the actual and predicted observations.

After (8) is whitened, it is finally transformed into a standard least squares optimization solution, as shown in (9).

(9) $$\begin{array}{c} \delta^{*}=\underset{\delta }{\arg\;\min}{\left\|A_{i}\delta_{i}-b_{i}\right\|}_{2}^{2}\\ =\underset{\delta }{\arg\;\min}{\left\|A\delta -b\right\|}_{2}^{2} \end{array}$$

where $A_{i}=\Sigma_{i}^{-1/2}H_{i}$, $b_{i}=\Sigma_{i}^{-1/2}(z_{i}-h_{i}(X_{i}^{0}))$.

Because the direct solution requires a large amount of computation, the QR or Cholesky decomposition method is used to accelerate the solution.

(10) $$Q^{T}A=\left[\begin{array}{c} R\\ 0 \end{array}\right]\;Q^{T}b=\left[\begin{array}{c} d\\ e \end{array}\right]\;{\left\|A\delta -b\right\|}_{2}^{2}={\left\|Q^{T}A\delta -Q^{T}b\right\|}_{2}^{2}={\left\|R\delta -d\right\|}_{2}^{2}+{\left\|e\right\|}_{2}^{2}$$

However, in practical applications, with the increase in system running time, the number of factor nodes in the algorithm gradually increases. When the optimal value of the state variable is solved each time, the Jacobian matrix J needs to be recalculated, and the QR decomposition is performed to obtain R, so as to perform the least squares optimization calculation. This batch solution method undoubtedly increases the calculation amount of the system and has a great impact on the real-time requirements of navigation and positioning.

**Figure 1:**
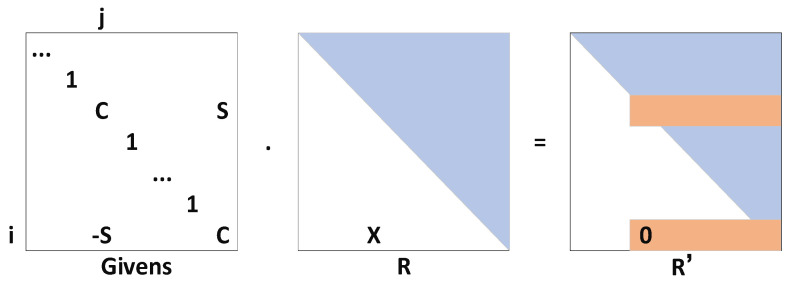
Givens matrix incremental update. (Using the Givens rotation matrix, a matrix is transformed into upper triangular form. Elements marked with X are eliminated, and elements marked with red are changed).

Through comparison, the literature [35,36,37] found that the newly added factor only affects the adjacent node variables, so the Givens matrix is introduced, as shown in Figure 1. The sparseness of R is optimized by the incremental update form and by considering different elimination orders, which reduces the calculation amount for the least squares solution and increases the real-time performance of the system.

## 3. Factor Graph Multi-Source Fusion Model Framework

The navigation system is actually a multi-variable control system, which is usually expressed in the form of state space, so that the multi-source fusion navigation system can also be modeled in the form of a factor graph model. When using the factor graph modeling method to solve navigation and positioning results such as position and attitude, the state equation and observation equation of the multi-source fusion navigation system can be expressed in the form of a factor graph.

In this paper, the IMU factor graph model is used as the main body. When sensor measurement information such as GNSS and VO is valid, the corresponding factor nodes are connected into the IMU factor graph model.

**Figure 2:**
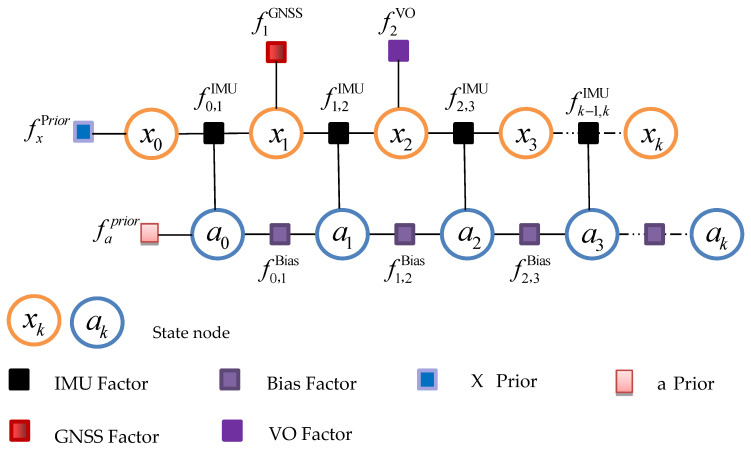
A multi-source fusion framework for the factor graph.

As shown in Figure 2, a factor graph architecture based on IMU/GNSS/VO is constructed, where x and a represent the state variable node, and f represents factors, including prior factors and measurement factors. $f_{x}^{Prior}$ and $f_{a}^{Prior}$ represent the prior factor of the state variable, $f_{k-1,k}^{IMU}$ represents the IMU pre-integration factor, $f_{k-1,k}^{Bias}$ represents the bias factor, and $f_{k}^{GNSS}$ and $f_{k}^{VO}$ represent the GNSS and VO measurement factors, respectively. x=[p,v,θ] represents the position, velocity, and attitude angle, respectively, and $a=[\omega_{a},a_{a}]$ represents the gyroscope bias and accelerometer bias, respectively.

### 3.1. IMU Pre-Integration Factor

The IMU measurement data in one update cycle $\Delta t=t_{k+1}-t_{k}$ are pre-integrated at the carrier coordinate system at time $t_{k}$, and the attitude, position, and velocity increments are obtained as (11).

(11) $$\left\{\begin{array}{c} \theta_{k+1}=\theta_{k}+H{\left(\theta_{k}\right)}^{-1}\omega_{k}^{b}\Delta t\\ p_{k+1}=p_{k}+v_{k}\Delta t+R_{k}a_{k}^{b}\frac{\Delta t^{2}}{2}\\ v_{k+1}=v_{k}+R_{k}a_{k}^{b}\Delta t \end{array}\right.\;H(\theta )=\overset{\propto }{\underset{k=0}{\Sigma }}\frac{{\left(-1\right)}^{k}}{(k+1)!}{\left[\theta \right]}_{\times }^{k}$$

where $R_{k}=\exp({\left[\theta_{k}\right]}_{\times })$ represents the rotation vector. Δt represents the pre-integration time interval.

The factor node is represented as the error function that needs to be minimized, and the IMU pre-integration factor node expression is as follows (12).

(12) $$f_{k,k+1}^{IMU}(x_{k+1},x_{k},a_{k})={\left\|e_{k,k+1}^{IMU}\right\|}_{\Sigma_{k,k+1}^{IMU}}^{2}={\left\|x_{k+1}-h(x_{k+1},x_{k},a_{k})\right\|}_{\Sigma_{k,k+1}^{IMU}}^{2}$$

where $h(x_{k+1,}x_{k},a_{k})$ represents the measurement function and $\Sigma_{k,k+1}^{IMU}$ represents the IMU pre-integration measurement noise covariance.

### 3.2. IMU Bias Factor

The IMU bias factor node expression is as follows (13).

(13) $$f_{k,k+1}^{Bias}(a_{k+1},a_{k})={\left\|e_{k,k+1}^{Bias}\right\|}_{\Sigma_{k,k+1}^{Bias}}^{2}={\left\|a_{k+1}-h(a_{k})\right\|}_{\Sigma_{k,k+1}^{Bias}}^{2}$$

where $h(a_{k})$ represents the measurement function and $\Sigma_{k,k+1}^{Bias}$ represents the IMU bias measurement noise covariance.

### 3.3. GNSS Factor

GNSS can provide the position and velocity information, and the measurements are expressed as (14).

(14) $$z_{k}^{GNSS}=\left[\begin{array}{c} p_{k}^{GNSS}\\ v_{k}^{GNSS} \end{array}\right]=\left[\begin{array}{c} p_{k}+n_{p}^{GNSS}\\ v_{k}+n_{v}^{GNSS} \end{array}\right]$$

where $p_{k}^{GNSS}$ and $v_{k}^{GNSS}$ represent the GNSS position and velocity measurements, respectively. $n_{p}^{GNSS}$ and $n_{v}^{GNSS}$ represent the position and velocity measurement noise, respectively.

The updated GNSS factor nodes at time $t_{k}$ are as follows (15).

(15) $$f_{k}^{GNSS}(x_{k})={\left\|e_{k}^{GNSS}\right\|}_{\Sigma_{k}^{GNSS}}^{2}={\left\|z_{k}^{GNSS}-h(x_{k})\right\|}_{\Sigma_{k}^{GNSS}}^{2}$$

where $h(x_{k})$ represents the measurement function and $\Sigma_{k}^{GNSS}$ represents the GNSS measurement noise covariance.

### 3.4. VO Factor

VO can provide the position and attitude information, and the measurements are expressed as follows (16).

(16) $$z_{k}^{VO}=\left[\begin{array}{c} p_{k}^{VO}\\ \theta_{k}^{VO} \end{array}\right]=\left[\begin{array}{c} p_{k}+n_{p}^{VO}\\ \theta_{k}+n_{\theta }^{VO} \end{array}\right]$$

where $p_{k}^{VO}$ and $\theta_{k}^{VO}$ represent the position and attitude measurements of VO, respectively. $n_{p}^{VO}$ and $n_{\theta }^{VO}$ represent the position and attitude measurement noise, respectively.

The updated VO factor node at time *t* is as follows (17).

(17) $$f_{k}^{VO}(x_{k})={\left\|e_{k}^{VO}\right\|}_{\Sigma_{k}^{VO}}^{2}={\left\|z_{k}^{VO}-h(x_{k})\right\|}_{\Sigma_{k}^{VO}}^{2}$$

where $h(x_{k})$ represents the measurement function and $\Sigma_{k}^{VO}$ represents the VO measurement noise covariance.

## 4. Federated Kalman Filter and Adaptive Federated Kalman Filter (AFKF)

**Figure 3:**
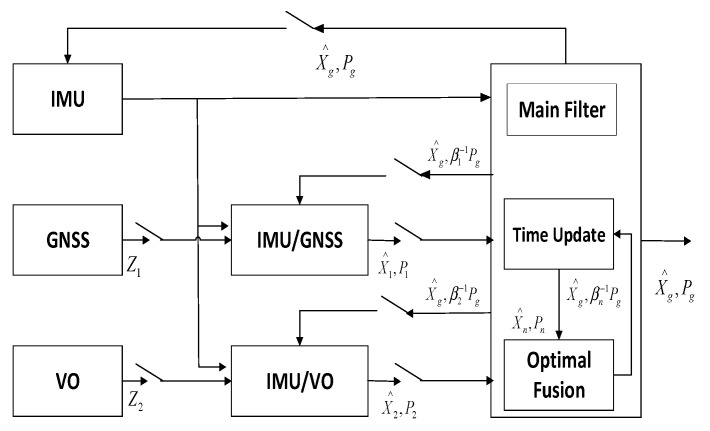
Federated Kalman filter.

**Figure 4:**
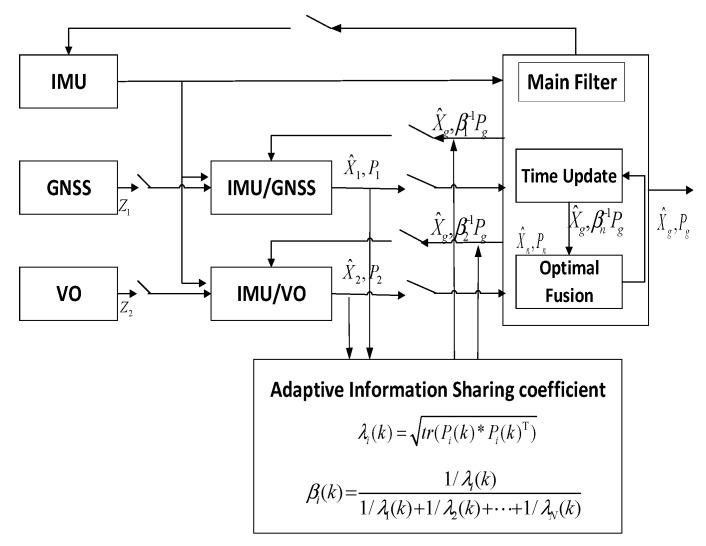
Adaptive federated Kalman filter.

In order to verify the applicability and robustness of the factor graph optimization multi-source fusion algorithm proposed in this paper, the traditional Kalman filter algorithm and the adaptive federated Kalman filter algorithm (cited from Ref. [38]) used for comparison are listed below, as shown in Figure 3 and Figure 4.

In Figure 4, the accuracy of the sub-filter $\lambda_{i}(k)$ is calculated by the state covariance $P_{i}$, as shown in (18).

(18) $$\lambda_{i}(k)=\sqrt{tr(P_{i}(k)*P_{i}{\left(k\right)}^{T})}$$

The adaptive information sharing coefficient $\beta_{i}(k)$ and sub-filter precision $\lambda_{i}(k)$ are expressed as follows:

(19) $$\beta_{i}(k)=\frac{1/\lambda_{i}(k)}{1/\lambda_{1}(k)+1/\lambda_{2}(k)+\cdots +1/\lambda_{N}(k)},i=1,2,\cdots ,N$$

Compared with the traditional Kalman filter algorithm, the adaptive Kalman filter algorithm in Figure 4 utilizes the information-sharing coefficients for adaptive allocation, thereby improving the robustness and accuracy of the entire system.

## 5. Simulation Experiment Verification

We specifically show here that, as in Ref. [38], the following mathematical simulation experiments in this paper are also developed and implemented on the basis of the PSINS toolbox and completed by Professor Yan Citizen of Northwestern Polytechnical University [39].

### 5.1. Trajectory Simulation Settings

**Figure 5:**
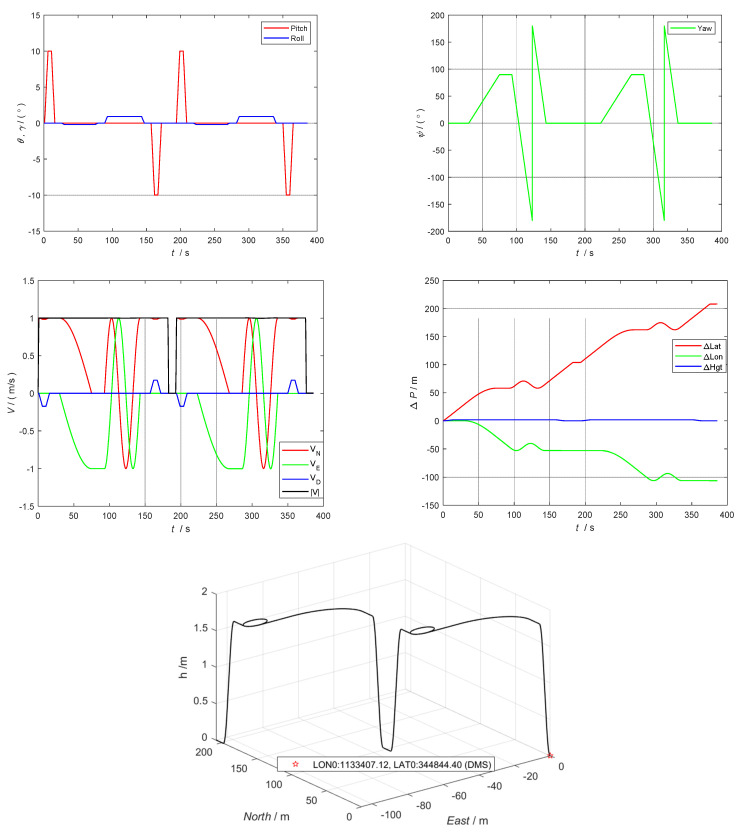
Trajectory and parameter state simulation.

According to the test requirements, the trajectory of the UAV is designed, with the initial position (longitude, latitude, and altitude) being 34.812332°, 113.568645°, and 0 m, respectively, and the initial attitude (pitch, roll, and yaw) being 0°, 0°, and 0°, respectively. The initial velocity (north, east, and ground) is 0, 0, and 0 m/s, respectively. The simulated UAV motion state includes acceleration, turning, climbing, descending, decelerating, etc. The trajectory and related position, speed, and attitude state quantities are shown in Figure 5.

### 5.2. Simulation Parameter Settings

According to the trajectory requirements, the parameters of the sensors configured by the UAV are set, as shown in Table 1.

### 5.3. Simulation Scene Settings

In this paper, the simulation scene is set in combination with the challenging environment faced by the UAV in actual flight, and the different state periods in which the sensor is prone to measurement errors are designed. The measurement errors and corresponding time periods produced by different sensors are shown in Table 2.

### 5.4. Experimental Results and Discussion

To verify the adaptability and robustness of the factor graph optimization algorithm, on the basis of a comparative analysis of the accuracy and time efficiency of different sliding windows, the results of the factor graph optimization algorithm, the traditional federated Kalman filtering algorithm, and the adaptive federated Kalman filtering algorithm were obtained for comparison and analysis.

#### 5.4.1. Comparative Analysis of FGO Sliding Window Size

The calculation time and accuracy of the FGO algorithms in this paper are tested. The test environment is Ubuntu 18.04, MATLAB 2018a; the test platform is configured using a 1.99 GHz, Intel(R) Core (TM) i7-8550U CPU. The test software is MATLAB 2018a, and the gtsam [40,41] library is used. By exploiting Cholesky decomposition and by constructing NonlinearFactorGraph using the Gaussian mixture model, the factor graph optimization model is built.

**Figure 6:**
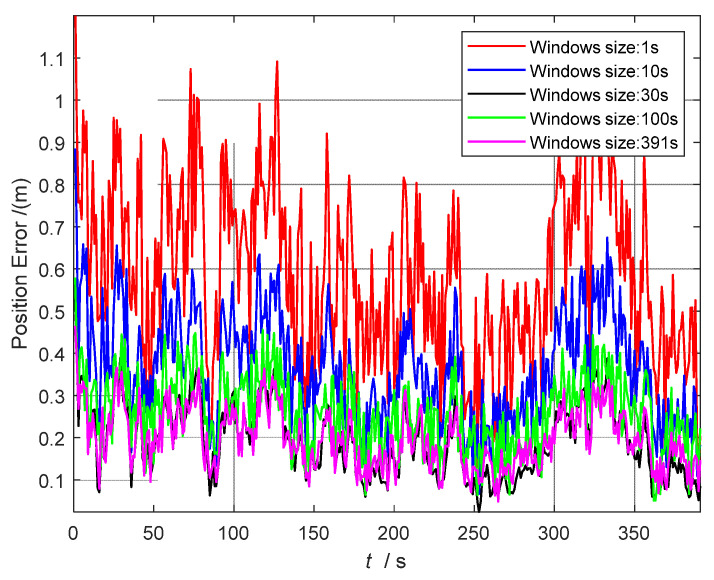
Variation in position error for different sliding window sizes, including 1, 10, 30, 100, and 391 s.

In order to compare and to analyze the relationship between real-time performance and accuracy, Figure 6 shows the change in position accuracy of different sliding window sizes, and Table 3 shows the comparison results of the position errors of different window size factor maps and the time used for the single-step execution.

As shown in Table 3, the accuracy continues to improve as the sliding window size increases. This is because the historical information is increased after the window is increased, especially for the window of 391, which is equivalent to batch optimization. Batch optimization considers global information, so that the accuracy is the highest. However, as the window increases, the usage time also increases, which is a major challenge for real-time performance. For a window larger than 30 s (100 s, 391 s), the positioning accuracy is not much improved, but the time consumption is significantly increased. For example, a single-step operation with a window of 391 takes twice as long as a window of 30 s, which exceeds the real-time requirement. Finally, considering the balance of time and accuracy, 30 s is chosen as the FGO sliding window size in the following.

#### 5.4.2. Track Comparison Results

In this study, three algorithms (FKF, AFKF, and FGO) were used for simulation experiments; the simulation trajectories, attitude, speed, and position errors were compared and analyzed; and the applicability of the FGO algorithm was analyzed.

**Figure 7:**
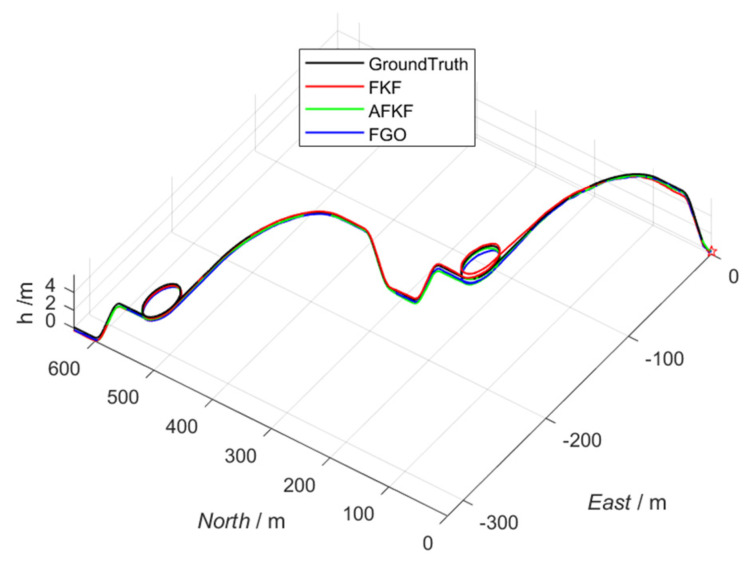
Comparison of different algorithm trajectories.

First, the simulation comparison of the trajectory is shown in Figure 7, below. Black lines represent true trajectories, red lines represent FKF, green lines represent AFKF, and blue lines represent FGO. It can be seen from Figure 7 that all three simulation algorithms can track the real trajectory, even in the two periods of sensor failure set in this paper. However, in general, the factor graph algorithm is closer to the real trajectory, followed by AFKF, and finally, FKF. This is because the factor graph method can realize the plug-and-play function simply by adding or subtracting the factor nodes corresponding to the navigation sensor. Compared with the adaptive federated Kalman filtering algorithm, the factor graph algorithm has better flexibility and scalability.

#### 5.4.3. Error Analysis and Precision Statistics

**Figure 8:**
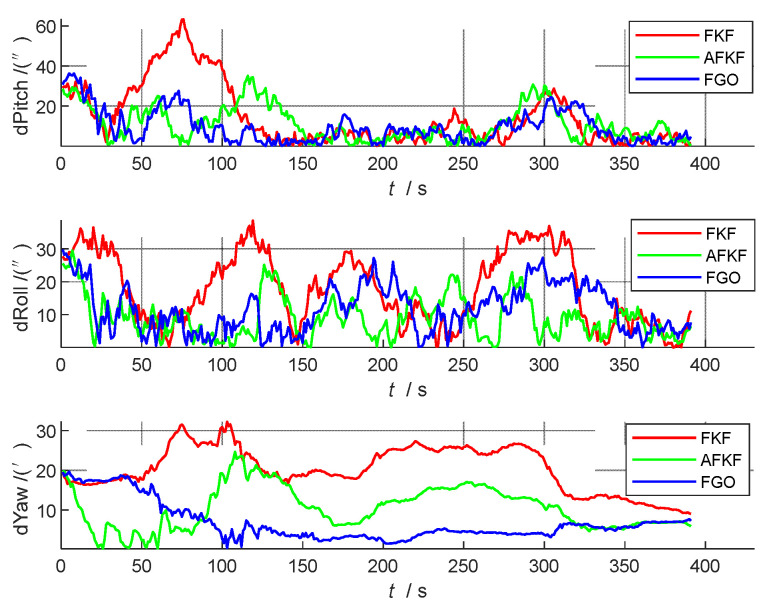
Comparison of attitude errors of different algorithms.

**Figure 9:**
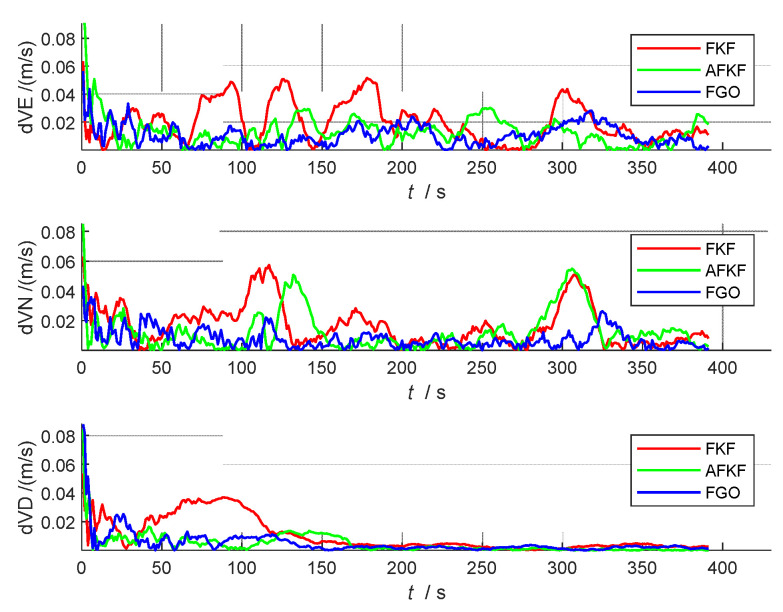
Comparison of speed errors of different algorithms.

**Figure 10:**
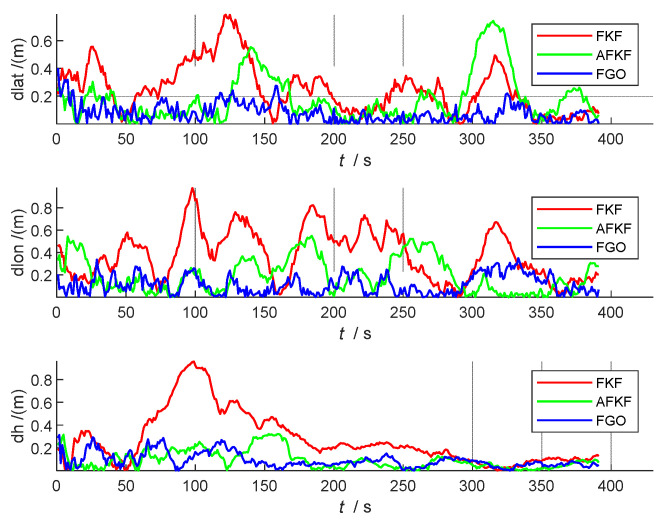
Comparison of position errors of different algorithms.

In order to further compare and analyze the effects of the three algorithms on the state quantity in detail, the following attitude errors (pitch angle, roll angle, and yaw angle), speed errors (north speed, east speed, and down speed), and position errors (latitude, longitude, and height) are compared, as shown in Figure 8, Figure 9 and Figure 10. The red line represents the FKF error result, the green line represents the AFKF error result, and the blue line represents the FGO error result.

From Figure 8, Figure 9 and Figure 10, compared with FKF, both AFKF and FGO have higher accuracies, and the advantage of position accuracy is more obvious. This is because both AFKF and FGO have good robustness, and they can maintain a good degree of accuracy, even in the presence of sensor gross errors and failures. However, compared with AFKF, FGO has a stronger stability and smaller errors in position, velocity, and attitude.

In order to quantitatively analyze the errors of each navigation parameter, the absolute mean error (AME), root mean square error (RMSE), and standard deviation (STD) of the three algorithms (FKF, AFKF, and FGO) are compared, as shown in Table 4. From the RMSE and AME data in Table 4, it can be seen that the state estimation result of FGO is the closest to the true value, and it can be seen from the STD data that the discrete degree of each state parameter of FGO is also small. Therefore, the FGO algorithm has the highest positioning accuracy, followed by AFKF, and finally FKF. Overall, the attitude of FGO is 8 arc minutes higher than that of FKF, and the speed is not much different, which is increased by 0.01 m/s, and the position is increased by 0.18 m.

#### 5.4.4. Statistical Analysis of Multiple Experiments

**Figure 11:**
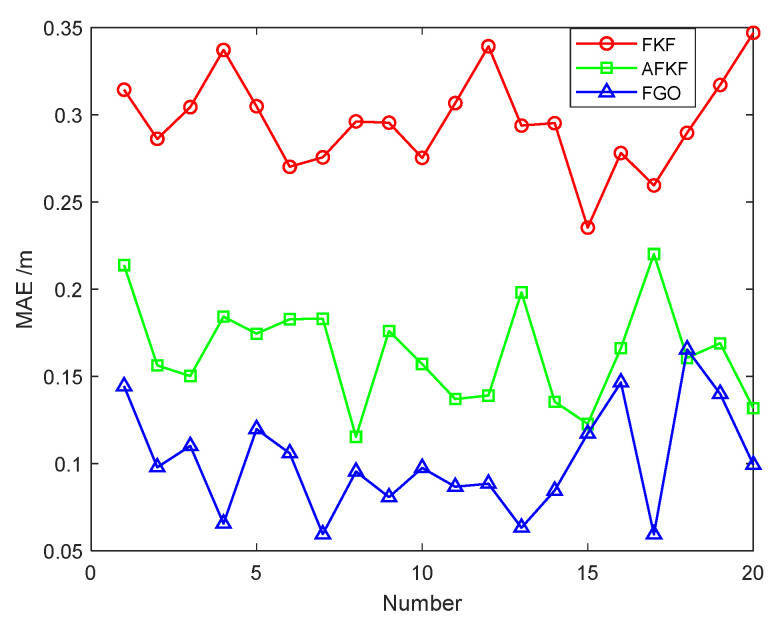
The MAEs of position errors (m) in 20 groups of Monte Carlo simulation experiments for the three algorithms (FKF, AFKF, and FGO).

To further compare the accuracies of the three algorithms, this study performed 20 groups of Monte Carlo simulations to simulate the real environment. The noise, trajectory, and speed of each setting are different. The mean absolute errors (MAEs) of the position errors for 20 groups of experiments are listed in Table 5, and the MAEs are shown in Figure 11.

As shown in Figure 11 and Table 2, the accuracy of FGO is significantly better than that of the other two algorithms. The average position error accuracies of the 20 groups of experiments were calculated separately, and the errors of the three algorithms are obtained as: 0.3013, 0.1598, and 0.0942 m. Compared with AFKF, the accuracy of FGO is increased by two-fold, and compared with FKF, the accuracy is increased by three-fold. The discussion and analysis of the above results further prove that the FGO algorithm proposed in this paper has a high accuracy and a good robustness, and the algorithm can be applied to complex environments.

## 6. Dataset Validation

**Figure 12:**
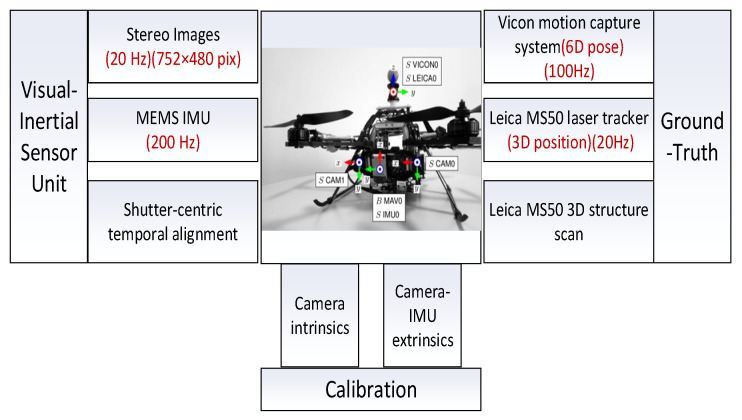
Asctec Firefly drone and sensor configuration parameters.

In order to further verify the applicability of the FGO algorithm, the following is verified by the EuRoC dataset [42]. The EuRoC dataset is a public dataset released by the Zurich University of Technology in 2016. The data collection platform is the Asctec Firefly, a rotary-wing drone, equipped with cameras, IMU, and visual motion capture systems. The specific structure and sensor configuration are shown in Figure 12, and the specific parameters and calibration data of the sensor are shown in Table 6.

The EuRoC data include both types of data: Industrial Machine Hall and Vicon Room. In order to fully verify the applicability of the algorithm in different scenarios and conditions, we chose three types of data: simple (MH_01_easy), medium (MH_03_medium), and more difficult (V1_03_difficult), for analysis and verification.

In order to verify the multi-source fusion effect of three sensors, IMU, VO, and GNSS, the GNSS data are simulated and generated according to the existing data (the location data of state_groundtruth_estimate0 is added with 0.5 m error, speed is added with 0.1 m/s error, and the frequency is 10 Hz). The VO data are calculated using ORB-SLAM2, and the GNSS and VO data are aligned for the following use. In order to verify the FGO algorithm in complex scenarios, errors are added to different sensors at different time periods, as shown in Table 7.

### 6.1. MH_01_Easy Scene

**Figure 13:**
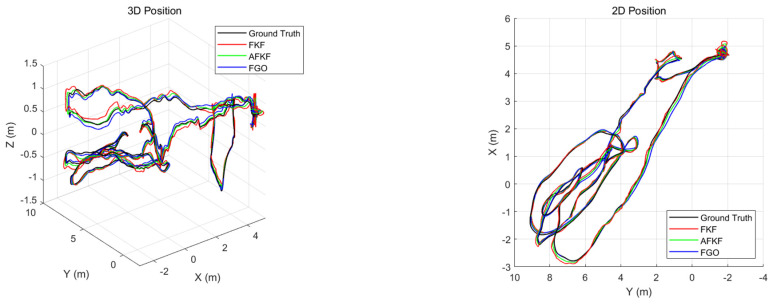
Comparison of 3D position and 2D position of different algorithms in MH_01_easy scene.

The MH_01_easy scene has a running length of 80.6 m and a duration of 182 s. It is a good texture and a bright scene. Figure 13 shows the 3D position graph and the 2D position graph of different algorithms for the MH_01_easy scene. The black line represents ground truth, the red line represents the state estimation result calculated by the FKF algorithm, the green line represents the state estimation result computed by the AFKF algorithm, and the blue line represents the state estimation result calculated by the FGO algorithm.

The 3D position and 2D position trajectories of the MH_01_easy scene are shown in Figure 13. Compared with AFKF and FKF, FGO is closer to the real trajectory, and has a smaller position error. However, the FGO trajectory appears to be locally unsmoothed, because the factor is automatically added according to different environments (sensor gross errors).

**Figure 14:**
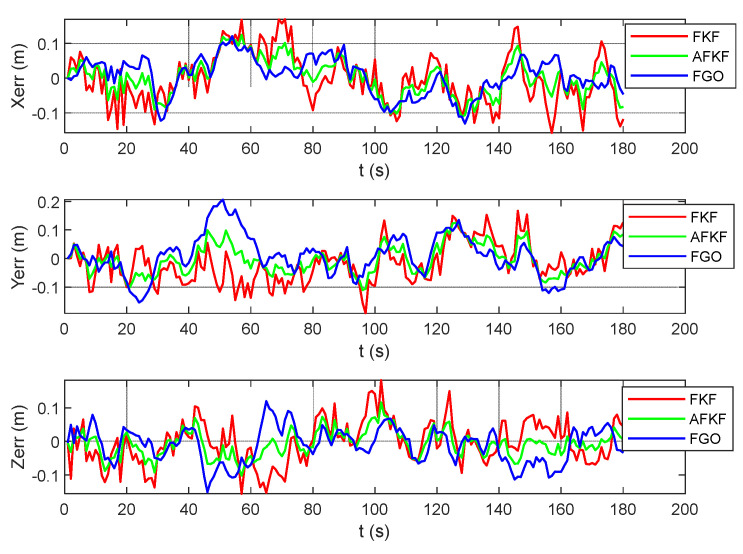
Comparison of position errors of different algorithms in MH_01_easy scene.

Figure 14 is the position error comparison diagram of the MH_01_easy scene. Red represents the FKF state estimation error, green represents the AFKF state estimation error, and blue represents the FGO state estimation error. FKF shows different changes according to the changes in different sensor errors in different time periods. The position error of FGO does not fluctuate, and the error value is small.

### 6.2. MH_03_Medium Scene

**Figure 15:**
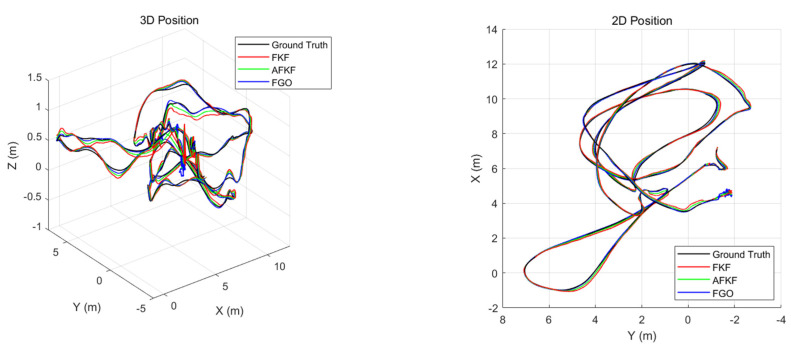
Comparison of 3D position and 2D position of different algorithms in MH_03_medium scene.

This scene has a running length of 130.9 m and a time of 132 s, and it is a fast-motion and bright scene. Similar to the MH_01_easy scene, Figure 15 shows a comparison diagram of the 3D position and 2D position of different algorithms for the MH_03_medium scene. The black, red, green, and blue lines, respectively, represent ground truth, FKF state estimation trajectory, AFKF state estimation trajectory, and FGO state estimation trajectory. As can be seen from Figure 15, the trajectories obtained by the three algorithms can all track the ground truth.

**Figure 16:**
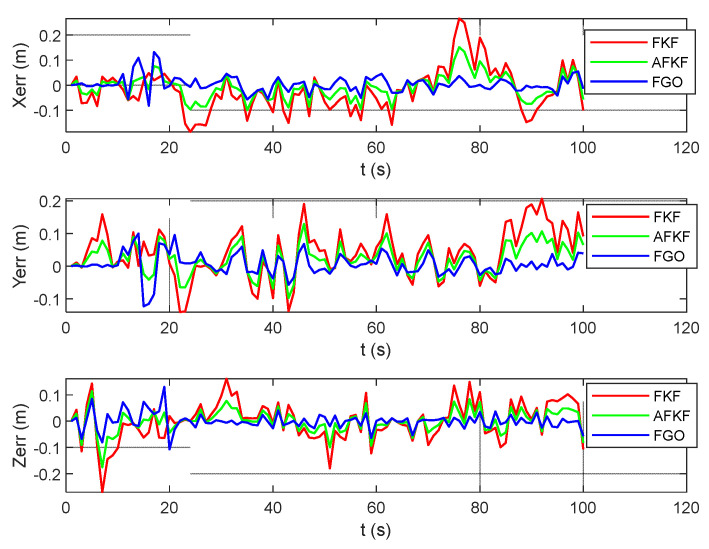
Comparison of position errors of different algorithms in MH_03_medium scene.

As shown in Figure 16, the error results for FKF, AFKF, and FGO are represented by red, green, and blue lines, respectively. Compared with the MH_01_easy scene, the position errors of the three algorithms in this scene are larger. However, compared with FKF and AFKF, FGO has a higher position accuracy.

### 6.3. V1_03_Difficult Scene

**Figure 17:**
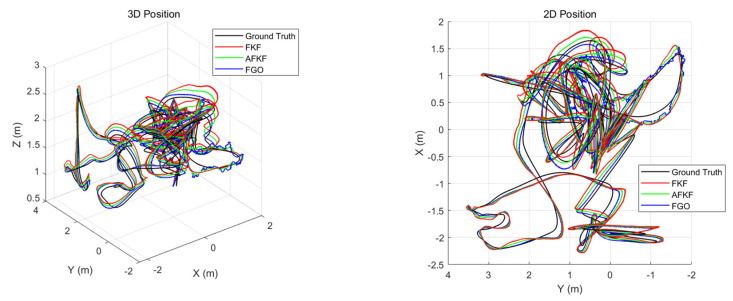
Comparison of 3D position and 2D position of different algorithms in V1_03_difficult scene.

**Figure 18:**
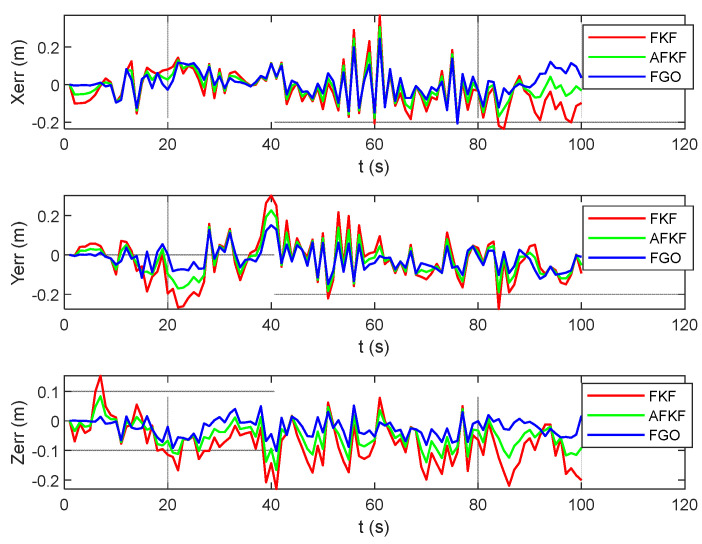
Comparison of position errors of different algorithms in V1_03_difficult scene.

The scene is a fast-motion and motion-blur scene, with a total running length of 79.0 m and a running time of 105 s. Similar to the MH_01_easy scene and the MH_03_medium scene, Figure 17 shows the 3D and 2D position comparison of different algorithms in the V1_03_difficult scene. The black, red, green, and blue lines represent ground truth, FKF state estimation trajectory, AFKF state estimation trajectory, and FGO state estimation trajectory, respectively. Figure 18 shows the error results for FKF, AFKF and FGO. Compared with the first two scenarios, the trajectories obtained by the FKF, AFKF, and FGO algorithms in this scenario have a certain deviation from the ground truth, but the effect of FGO is still better than that of AFKF and FKF.

To sum up the comparison, the data of the three scenarios are compared and analyzed, and the comparison results of the position error and the attitude errors of different algorithms in different scenarios are shown in Table 8. For a simple scene (MH_01_easy scene), FGO has obvious advantages. The algorithm has higher overall accuracy, and the accuracy remains relatively stable. As the complexity of the scene increases from the MH_01_easy scene to the V1_03_difficult scene, the overall accuracies of the FKF, AFKF, and FGO algorithms decrease, but the accuracy of FGO is still higher than those of AFKF and FKF. Combining the three scenarios, the position accuracy is obviously improved. Compared with AFKF and FKF, the positioning accuracy of FGO is improved by 1.5–2-fold. This positioning accuracy result is worse than the simulation data. This is because the difficulty of the scene increases, resulting in data with stronger dynamics, fewer visual feature points, and a larger range of motion.

## 7. Conclusions

To improve the reliability and robustness of UAV autonomous navigation and positioning in complex scenarios, we used a novel multi-source fusion algorithm framework for autonomous navigation and localization. The main innovation is that the multi-source fusion framework established in this paper considers the IMU pre-integration factor and IMU bias factor at the same time, and iSAM is used to solve it. The requirements of positioning accuracy and real-time performance can be met at the same time. In addition, compared with previous studies, the selection of the sliding window size was first considered in this paper. Compared with the traditional Kalman filter and the adaptive Kalman filter, the results show that FGO has the highest accuracy, followed by AFKF, and the worst is FKF. Finally, three scenarios of EuRoC datasets with different difficulties were compared and analyzed, which further verifies the usability and robustness of FGO. Combined with the mathematical simulation experiments and data set verification, compared with the other two algorithms, the position accuracy of FGO is improved by 1.5–2-fold.

In summary, the FGO algorithm in this paper can significantly improve the accuracy and tolerance of the navigation system in complex environments. However, so far, most factor graph algorithms are only in the experimental simulation stage, are rarely applied to practical systems [43,44,45], and a lot of theoretical work and engineering practice are needed in the next step. The multi-sensor fusion algorithm based on a factor graph in this paper is based on a loosely coupled structure. In the next research, we plan to establish a tightly coupled fusion framework to further improve the navigation accuracy.

## Figures and Tables

**Table 1:** Sensor parameter settings.

Sensor Type	Parameter		Value
IMU	Gyro error (x-, y-, z-)	bias	$0.2^{{}^{\circ}}/h$
		random walk	$0.08^{{}^{\circ}}/\sqrt{h}$
	Accelerometer error (x-, y-, z-)	bias	100 μg
		random walk	$20\;\mu g/\sqrt{h}$
	Frequency		100 Hz
GNSS	Location (longitude, latitude, altitude)		[1 m; 1 m; 2 m]
	Speed (north, east, down)		[0.1 m/s; 0.1 m/s; 0.1 m/s]
	Frequency		1 Hz
VO	Location (x-, y-, z-)		[0.5 m; 0.5 m; 0.5 m]
	Attitude (pitch-, yaw-, roll-)		[0.5°; 0.5°; 0.5°]
	Frequency		1 Hz

**Table 2:** Measurement errors of different sensors at different time periods (40~170 s, 20 times the $\Sigma_{k}^{GNSS}$ gross error is added to the GNSS positioning measurement. In the range of 410~500 s, 10 times the $\Sigma_{k}^{VO}$ gross error is added to the VO positioning measurement).

Sensor Type	40~170 s	410~500 s
VO		$10\;\times \;\Sigma_{k}^{VO}$
GNSS	20 × $\Sigma_{k}^{GNSS}$	

**Table 3:** Comparison results of position accuracy and the time used for the single-step execution for different window sizes.

**Windows Size (s)**	1	10	30	100	391
**Position Error (m)**	0.24	0.17	0.12	0.10	0.09
**The Used Time (s)**	5.6971 × 10^−2^	6.9302 × 10^−2^	7.4211 × 10^−2^	9.8083 × 10^−2^	1.23402 × 10^−1^

**Table 4:** Error statistics of different algorithms.

Error		Dlat (m)	Dlon (m)	dH (m)	dVN (m/s)	dVE (m/s)	dVD (m/s)	Dpith (′)	Dyaw (′)	Droll (′)
FKF	AME	0.25	0.37	0.27	0.02	0.02	0.01	14.55	17.66	20.33
	RMSE	0.31	0.44	0.36	0.02	0.02	0.02	20.91	20.70	21.07
	STD	0.18	0.22	0.23	0.06	0.06	0.02	19.47	19.18	15.53
AFKF	AME	0.18	0.19	0.09	0.01	0.01	0.00	11.06	8.98	10.43
	RMSE	0.25	0.25	0.12	0.02	0.02	0.01	13.86	10.93	11.67
	STD	0.17	0.15	0.08	0.03	0.05	0.02	13.90	10.98	6.68
FGO	AME	0.08	0.11	0.09	0.01	0.01	0.01	9.33	11.59	6.81
	RMSE	0.10	0.14	0.11	0.01	0.01	0.01	12.31	13.52	8.31
	STD	0.07	0.08	0.06	0.01	0.01	0.01	12.34	11.60	3.99

**Table 5:** The MAE values of position errors (m) in 20 groups of Monte Carlo simulation experiments for the three algorithms (FKF, AFKF, and FGO).

Number	FKF	AFKF	FGO
1	0.3144	0.2138	0.1444
2	0.2862	0.1563	0.0979
3	0.3044	0.1502	0.1101
4	0.3372	0.1843	0.0656
5	0.3049	0.1745	0.1198
6	0.2702	0.1828	0.1059
7	0.2756	0.1831	0.0595
8	0.2962	0.1154	0.0954
9	0.2955	0.1760	0.0807
10	0.2753	0.1571	0.0975
11	0.3067	0.1370	0.0867
12	0.3393	0.1390	0.0885
13	0.2938	0.1982	0.0632
14	0.2952	0.1355	0.0845
15	0.2353	0.1227	0.1172
16	0.2781	0.1662	0.1465
17	0.2595	0.2201	0.0594
18	0.2897	0.1606	0.1655
19	0.3171	0.1690	0.1400
20	0.3470	0.1317	0.0993

**Table 6:** Calibration values of internal and external parameters of the sensor (reprinted/adapted with permission from Ref. [42]).

Parameter		Value
Camera	Resolution	[752, 480] pix
	intrinsics	$\left[\begin{array}{ccc} 458.654 & 0 & 367.215\\ 0 & 457.296 & 248.375\\ 0 & 0 & 1 \end{array}\right]$
	distortion_coefficients	$\left[\begin{array}{cccc} -0.28340811 & 0.07395907 & 0.00019359 & 1.76187114\times 10^{-5} \end{array}\right]$
IMU	gyroscope_noise_density	$1.6968\times 10^{-4}\;\mathrm{rad}/s/\sqrt{\mathrm{Hz}}$
	gyroscope_random_walk	$1.9393\times 10^{-5}\;\mathrm{rad}/s^{2}/\sqrt{\mathrm{Hz}}$
	accelerometer_noise_density	$2.0000\times 10^{-3}\;{m/s}^{2}/\sqrt{\mathrm{Hz}}$
	accelerometer_random_walk	$3.0000\times 10^{-3}\;{m/s}^{3}/\sqrt{\mathrm{Hz}}$
Camera-IMU extrinsics	$\left[\begin{array}{cccc} 0.0148655429818 & -0.999880929698 & 0.00414029679422 & -0.0216401454975\\ 0.999557249008 & 0.0149672133247 & 0.025715529948 & -0.064676986768\\ -0.0257744366974 & 0.00375618835797 & 0.999660727178 & 0.00981073058949\\ 0.0 & 0.0 & 0.0 & 1.0 \end{array}\right]$	

**Table 7:** Different sensor simulation error parameter values.

Scenes	Sensor Type	Value	Period
MH_01_easy	GNSS	$20\times \Sigma_{k}^{GNSS}$	40–100 s
	VO	$10\times \Sigma_{k}^{VO}$	140–180 s
MH_03_medium	GNSS	$20\times \Sigma_{k}^{GNSS}$	20–40 s
	VO	$10\times \Sigma_{k}^{VO}$	60–90 s
V1_03_difficult	GNSS	$20\times \Sigma_{k}^{GNSS}$	20–40 s
	VO	$10\times \Sigma_{k}^{VO}$	70–90 s

**Table 8:** Accuracy comparison results of different algorithms in different scenarios.

Experimental Scene	Algorithm	Position Error (m)		
		x	y	z
MH_01_easy	FKF	0.07	0.07	0.07
	AFKF	0.06	0.06	0.05
	FGO	0.04	0.04	0.03
MH_03_medium	FKF	0.09	0.08	0.07
	AFKF	0.07	0.06	0.05
	FGO	0.05	0.05	0.03
V1_03_difficult	FKF	0.12	0.14	0.11
	AFKF	0.10	0.11	0.08
	FGO	0.09	0.09	0.05

## Data Availability

Not applicable.
